# Supplementary material for: Bacterial cyclic diguanylate signaling networks sense temperature
Source: Nat Commun. 2021 Mar 31;12:1986. doi: 10.1038/s41467-021-22176-2 (PMC8012707; doi:10.1038/s41467-021-22176-2)
Supplement: Supplementary file 1 — Supplementary Information [file 41467_2021_22176_MOESM1_ESM.pdf]

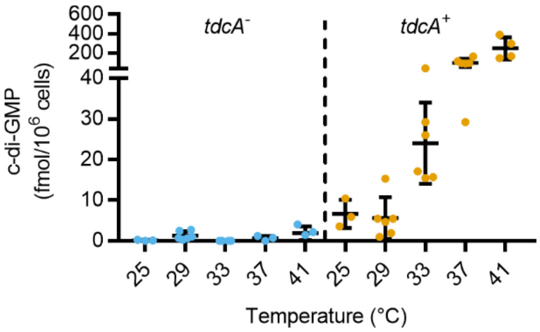


**Supplementary Figure 1. Temperature-dependent synthesis of c-di-GMP in *P. aeruginosa* PAO1 expressing *tdcA*.** Liquid chromatography tandem mass spectrometry (LC-MS/MS) was used to measure c-di-GMP in cellular nucleotide pools. Each datum point represents an independent biological replicate. Lines and bars represent the means and standard deviations for three to six independent biological replicates. The *tdcA*$^{-}$ and *tdcA*$^{+}$ strains are represented by sky blue and orange dots, respectively. The strain denoted *tdcA*$^{-}$ has the *tdcA*$_{162\Delta G}$ allele.

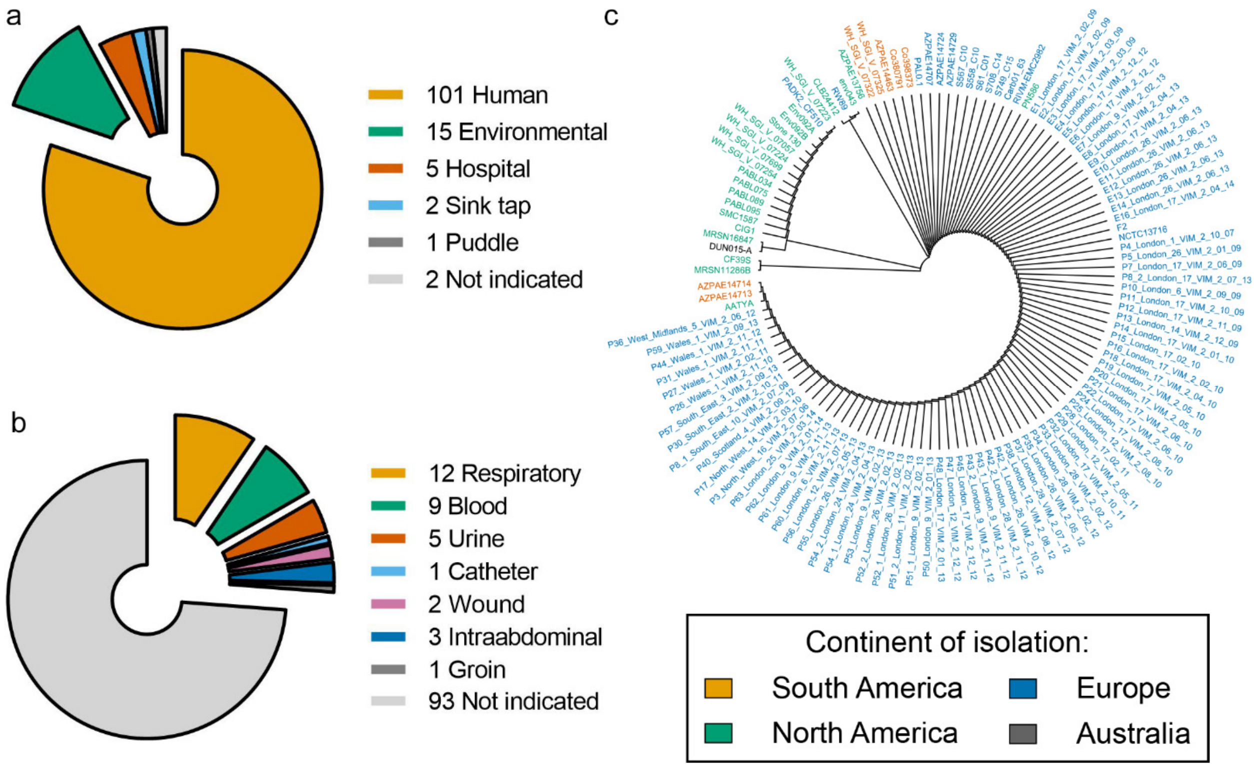


**Supplementary Figure 2. The *tdcA* gene is found in genome sequences of human and environmental *Pseudomonas aeruginosa* strains that have been isolated worldwide.** (a) Isolation source and (b) sample type taken from genome metadata for 126 *P. aeruginosa* strains found to encode a tdcA allele (colored by isolation source and sample type, respectively, see graphical legends). In (a), isolation sources are color coded orange for human, bluish green for environmental, vermillion for hospital, sky blue for sink tap, dark grey for puddle, and light grey for not indicated. In (b), sample types are color coded orange for respiratory, bluish green for blood, vermillion for urine, sky blue for catheter, reddish purple for wound, blue for intraabdominal, dark grey for groin, and light grey for not indicated. The respiratory sample type includes cystic fibrosis sputum, respiratory tract, and pneumonia isolates. (c) Unrooted Jukes-cantor genetic distance model of tdcA alleles in 119 P. aeruginosa strains (colored by continent of isolation: orange for South America, bluish green for North America, blue for Europe, and dark grey for Australia; see graphical legend).

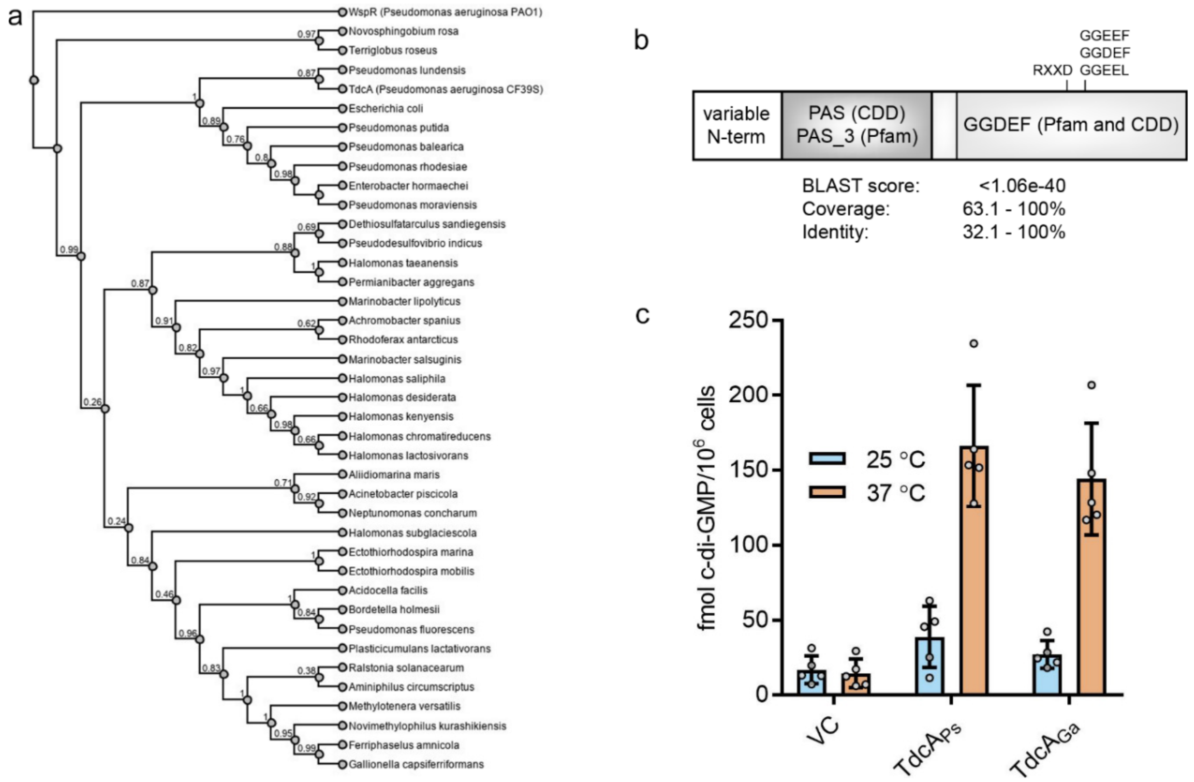


**Supplementary Figure 3. TdcA homologs are distributed throughout bacterial genomes.** (a) FastTree2 maximum-likelihood dendrogram illustrating the phylogenetic distributions of 39 high-confidence TdcA orthologues. The *P. aeruginosa* WspR sequence was chosen as an outgroup based on related enzymatic function but non-identity of the CheY sensory domain with the TdcA thermoPAS domain. Values represent FastTree support values for each of the nodes. (b) Features and InterProScan 5 [ref. [1]] domain function classification of TdcA homologs. CDD, Conserved Domain Database; Pfam, Protein Family database. (c) LC-MS/MS measurements of c-di-GMP levels in cells expressing a vector control (VC), *P. aeruginosa* CF39S TdcA ($TdcA_{Ps}$) or the *Gallionellales* bacterium GWA2_54_124 TdcA homolog ($TdcA_{Ga}$, NCBI Accession No. OGS67013.1, https://www.ncbi.nlm.nih.gov/protein/OGS67013.1) from an arabinose inducible expression cassette (*araC-$P_{BAD}$*) in *P. aeruginosa* PAO1. Each datum point represents an independent biological replicate, and bars and lines represent means and standard deviations, respectively, for 5 independent biological replicates. Temperatures of 25 °C and 37 °C are represented by sky blue and orange bars, respectively.

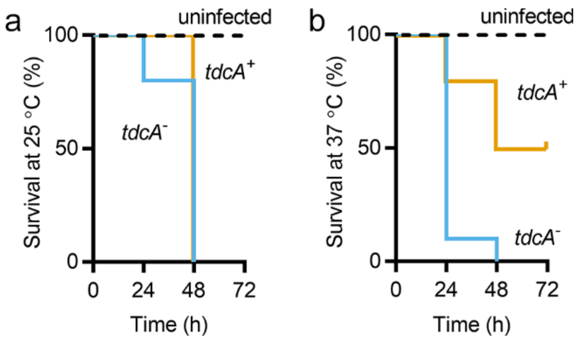


**Supplementary Figure 4. The thermosensory diguanylate cyclase (TdcA) orchestrates temperature-dependent *P. aeruginosa* virulence in waxworms.** (a and b) Temperature-dependent lethality in *Galleria mellonella* at (a) 25 °C and (b) 37 °C. Data represent counts for three independent groups of ten waxworms. Survival is significantly different between PAO1 *tdcA*$^+$ and PAO1 *tdcA*$^-$ (*tdcA*$_{162\Delta G}$) strains ($P < 0.0001$) after 72 h at 37 °C by means of a Mantel-Cox log-rank test. By contrast, no significant difference was observed between PAO1 *tdcA*$^+$ and PAO1 *tdcA*$^-$ strains at 25 °C ($P < 0.1462$). The *tdcA*$^-$ and *tdcA*$^+$ strains are represented by sky blue and orange lines, respectively. The strains denoted *tdcA*$^-$ have the *tdcA*$_{162\Delta G}$ allele.

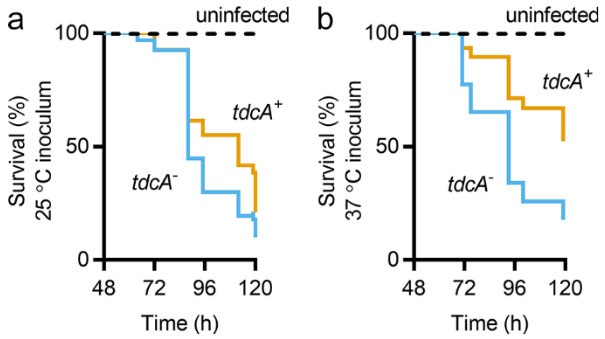


**Supplementary Figure 5. The thermosensory diguanylate cyclase (TdcA) is linked to temperature-dependent attenuation of *P. aeruginosa* CF39S virulence in *Caenorhabditis elegans*.** (a) *C. elegans* survival when inoculum is prepared at 25 °C. (b) *C. elegans* survival when inoculum is prepared at 37 °C. Survival is significantly different between CF39S (*tdcA*$^{+}$) and CF39 (*tdcA*$^{-}$) strains after 120 h at both 25 °C ($P$ = 0.0038) and 37 °C ($P$ < 0.0001) by means of a Mantel-Cox log-rank test. However, there is a significantly greater proportion of nematodes that survive infection with CF39S (*tdcA*$^{+}$) at when the inoculum is prepared at 37 °C vs. 25 °C ($P$ < 0.0001), whereas no significant difference was observed for CF39 (*tdcA*$^{-}$) prepared under the same conditions ($P$ = 0.7221). The *tdcA*$^{-}$ and *tdcA*$^{+}$ strains are represented by sky blue and orange lines, respectively. Strains denoted *tdcA*$^{-}$ have the *tdcA*$_{162\Delta G}$ allele.

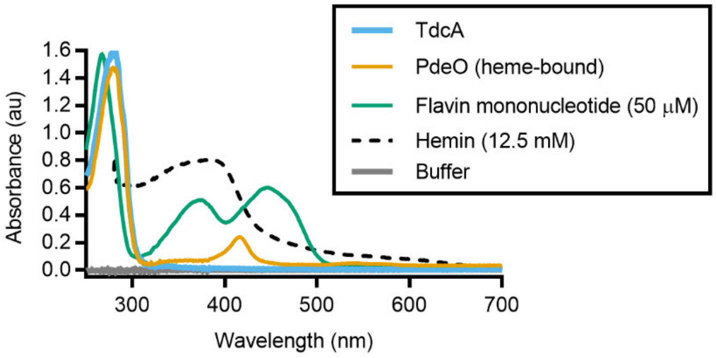


**Supplementary Figure 6. TdcA does not display a spectral signature for heme or flavin cofactors.** UV-Vis spectrophotometry of purified recombinant 6×His-MBP-TdcA and DosP (a heme-binding protein, also called PdeO), as well as hemin and flavin mononucleotide (FMN) standards (au denotes arbitrary units). Solid lines represent means of five technical replicates for the purified, recombinant proteins or cofactor (sky blue for TdcA, orange for PdeO, bluish green for flavin mononucleotide, and grey for the buffer control). The hatched black line represents a single technical replicate for hemin.

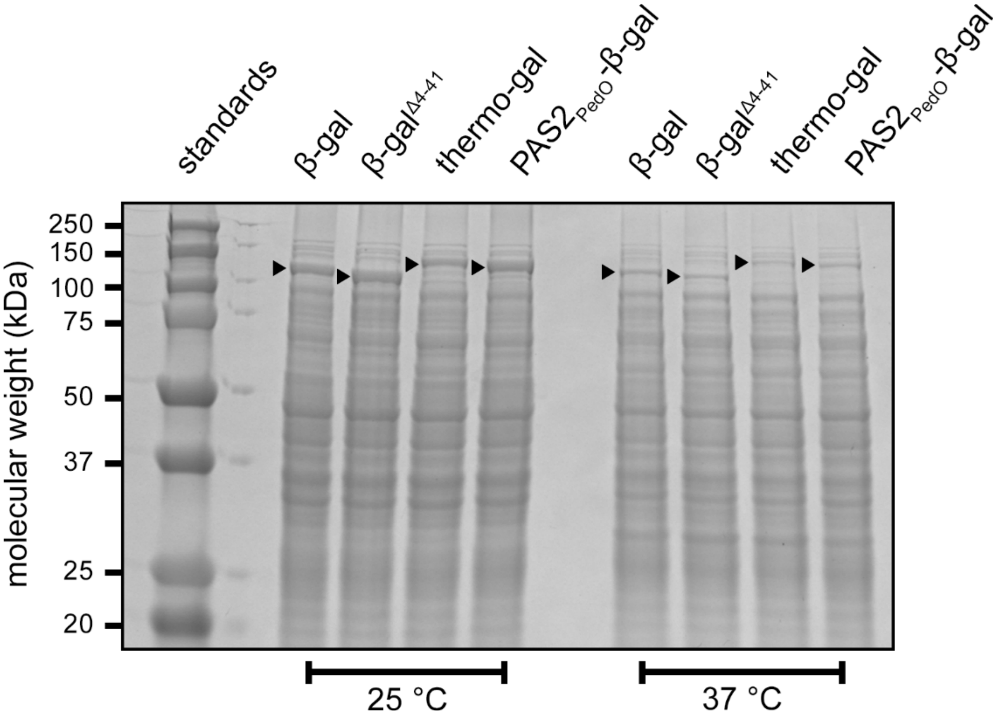


**Supplementary Figure 7. β-galactosidase and the synthetic protein thermo-gal are similarly abundant at both 25 °C and 37 °C when expressed in *E. coli* LMG194.** SDS-PAGE gels were loaded with 15 µg of total cellular protein per lane. Arrows indicate a protein band corresponding to the molecular weight of the indicated heterologously expressed protein. A representative gel image from a single batch purification of protein from *Escherichia coli* is shown here, which was executed once as a standard practice for qualitative assessment of recombinant protein expression levels in different strains.

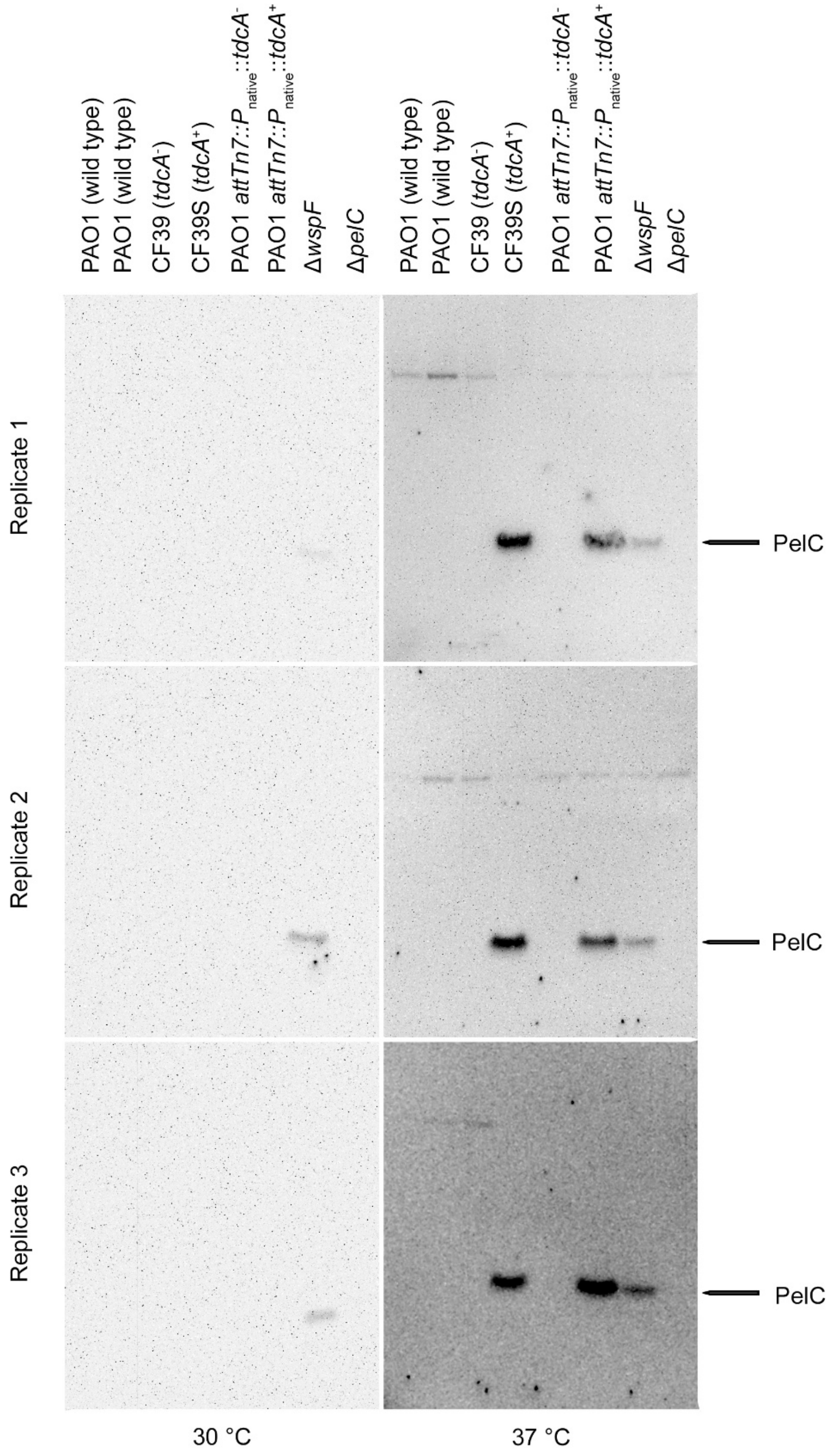


**Supplementary Figure 8. Original chemiluminescent images of Western blots for PelC using anti-PelC antiserum.** Each lane was loaded with an equivalent, standardized quantity of total cellular protein.

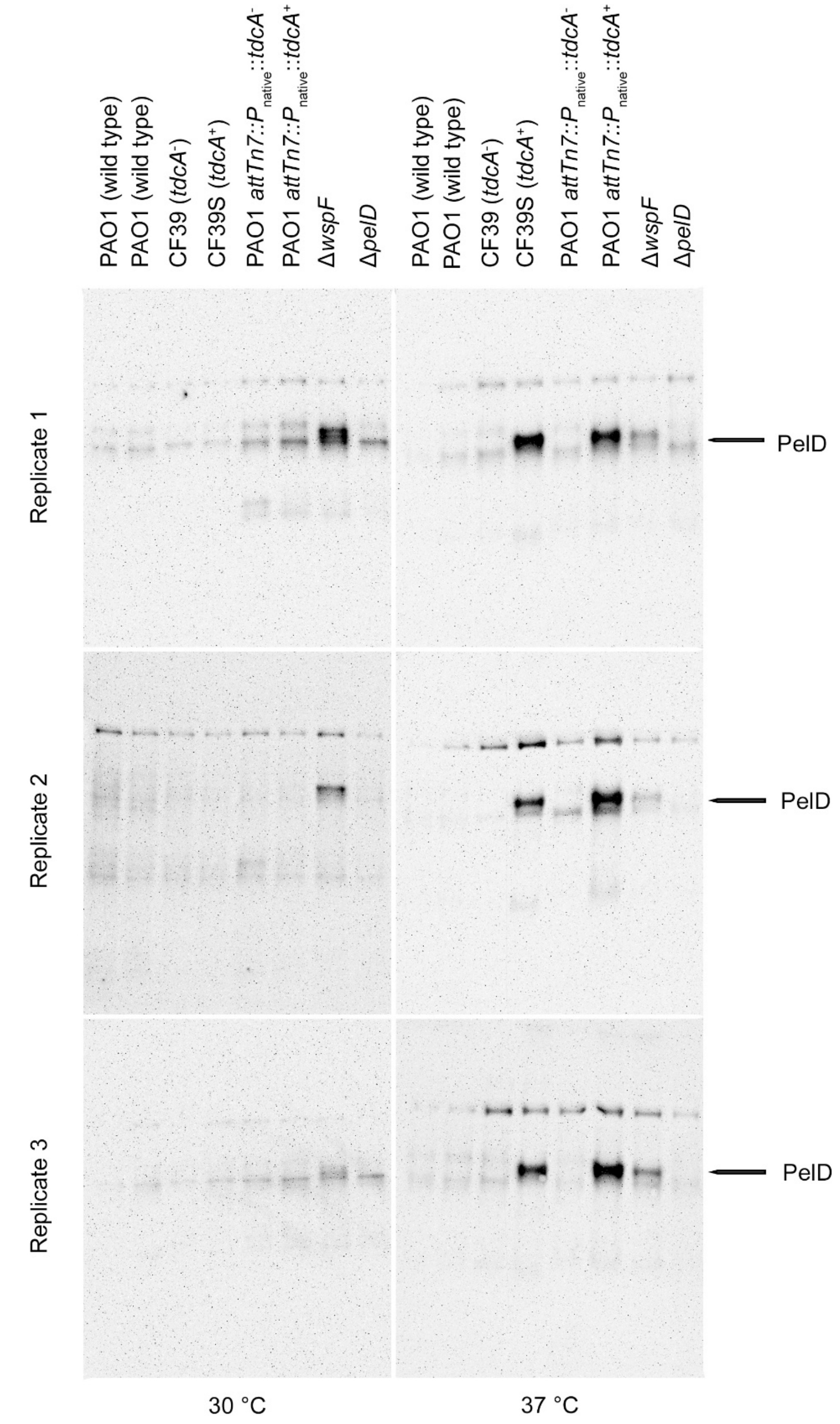


**Supplementary Figure 9. Original chemiluminescent images of Western blots for PelD using anti-PelD antiserum.** Each lane was loaded with an equivalent, standardized quantity of total cellular protein.

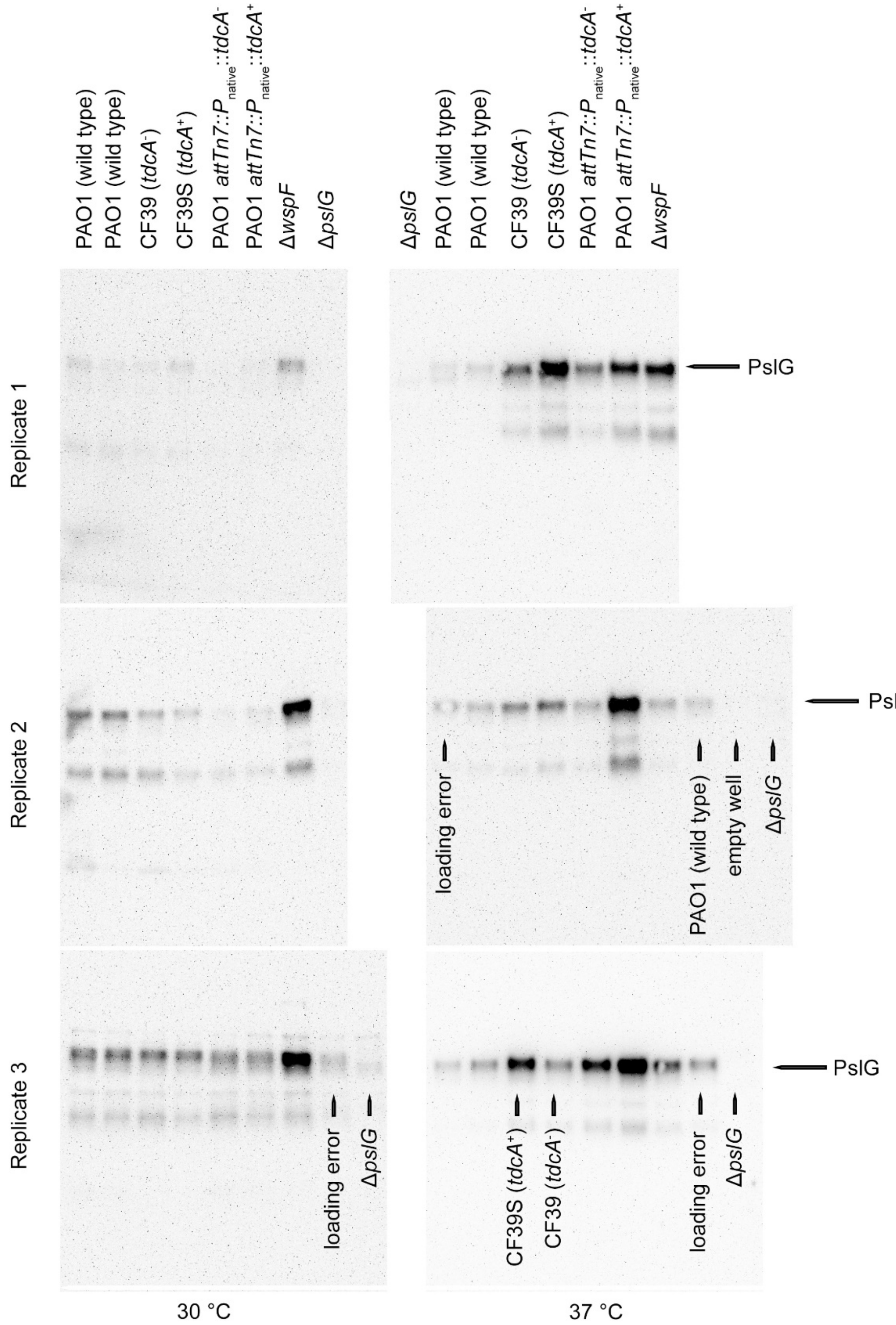


**Supplementary Figure 10. Original chemiluminescent images of Western blots for PslG using purified anti-PslG antibodies.** Each lane was loaded with an equivalent, standardized quantity of total cellular protein. Lanes loaded differently than the standard order labelled at top are marked individually.

**Supplementary Table 1 – Bacterial strains.**

| Strain | Genotype, description or relevant characteristics [a] | Source |
|---|---|---|
| *Pseudomonas aeruginosa* | | |
| CF39 | Clinical isolate from a cystic fibrosis patient ($tdcA_{162\Delta G}$) | [2] |
| CF39S | Clinical isolate from a cystic fibrosis patient (*tdcA*) | [2] |
| LS28 | CF39S with $tdcA_{162\Delta G}$ from CF39 | This study |
| LS36 | CF39 with *tdcA* from CF39S | This study |
| TER39-1E | CF39 Δ*pelF* | This study |
| TER43-4G | CF39 Δ*pelF* Δ*pslD* | This study |
| TER40-2G | CF39S Δ*pelF* | This study |
| TER44-6A | CF39S Δ*pelF* Δ*pslD* | This study |
| TER53-A | CF39 Δ*fleQ* | This study |
| TER54-A | CF39S Δ*fleQ* | This study |
| JJH0 | PAO1 wild type strain originating from the laboratory of Dr. Colin Manoil (MPAO1)[3], genome re-sequenced in-house | [4] |
| TER1 | JJH0 *att*Tn7::miniTn7T2.1-Gm-GW::$P_{native}$::*tdcA* | This study |
| KL5 | JJH0 *att*Tn7::miniTn7T2.1-Gm-GW::$P_{native}$::$tdcA_{162\Delta G}$ | This study |
| JJH863 | JJH0 *att*Tn7:miniTn7T2-Gm-GW bearing the putative 7-gene operon with *tdcA* from *P. aeruginosa* CF39S | This study |
| KL4 | JJH0 *att*Tn7:miniTn7T2-Gm-GW bearing the putative 7-gene operon with $tdcA_{162\Delta G}$ from *P. aeruginosa* CF39 | This study |
| JJH283 | JJH0 Δ*fliC* | [4] |
| JJH389 | JJH0 Δ*fleQ* | [4] |
| TER57-A | JJH389 *att*Tn7::miniTn7T2.1-Gm-GW::$P_{native}$::*tdcA* | This study |
| TER58-A | JJH389 *att*Tn7::miniTn7T2.1-Gm-GW::$P_{native}$::$tdcA_{162\Delta G}$ | This study |
| JJH485 | JJH0 Δ*pelF* | [5] |
| JJH498 | JJH0 Δ*pslD* | [6] |

| | | |
|---|---|---|
| JJH502 | JJH0 Δ*pelF* Δ*pslD* | [7] |
| HAL12 | JJH502 *att*Tn7::miniTn7T2.1-Gm-GW::$P_{native}$::*tdcA*, $Gm^r$ | This study |
| HAL08 | JJH502 *att*Tn7: $P_{native}$::*tdcA*, $Gm^s$ | This study |
| HAL11 | JJH502 *att*Tn7::miniTn7T2.1-Gm-GW::$P_{native}$::*tdcA*$_{162\Delta G}$, $Gm^r$ | This study |
| HAL07 | JJH502 *att*Tn7::$P_{native}$::*tdcA*$_{162\Delta G}$, $Gm^s$ | This study |
| HAL162 | JJH502 *att*Tn7::miniTn7T-Gm, $Gm^r$ | This study |
| HAL163 | JJH502 *att*Tn7::miniTn7T-Gm*::araC-*$P_{BAD}$*::wspR*$^{V72D}$, $Gm^r$ | This study |
| HAL164 | JJH502 *att*Tn7::miniTn7T-Gm*::araC-*$P_{BAD}$*::tdcA*, $Gm^r$ | This study |
| HAL165 | JJH502 *att*Tn7::miniTn7T-Gm*::araC-*$P_{BAD}$*::stp1*, $Gm^r$ | This study |
| *Pseudomonas fluorescens* | | |
| Pf-81 | Wild type | Howard Ceri |
| HAL81 | Pf-81 *att*Tn7::miniTn7T2.1-Gm-GW::$P_{native}$::*tdcA*$_{162\Delta G}$, $Gm^r$ | This study |
| HAL82 | Pf-81 *att*Tn7::miniTn7T2.1-Gm-GW::$P_{native}$::*tdcA*, $Gm^r$ | This study |
| *Escherichia coli* | | |
| K12 MG1655 | Wild type strain | Coli Genetic Stock Center |
| NEB5α | Strain for cloning | New England Biolabs |
| XL1-Gold | Strain for cloning | Agilent Technologies |
| NiCo21(DE3) | Strain for protein expression | New England Biolabs |
| S17.1 (λpir) | Donor strain, $Sm^r$ , $Tc^r$ | [8] |
| *ccdB* Survival 2™ T1R | Strain for cloning, $Str^r$ | Invitrogen |
| LMG194 | ATCC 47090, $F^-$ Δ(*lacIPOZY*)X74 *galE galK thi rpsL* Δ*phoA ara714* | American Type Culture Collection |
| Rosetta2™ (DE3) | Strain for protein expression, $Cm^r$ | Novagen |

[a]Abbreviations for antibiotic selection: Ap, ampicillin; Cb, carbenicillin; Cm, chloramphenicol, Gm, Gentamicin; Sm, streptomycin; Tc, tetracycline.

**Supplementary Table 2 – Plasmids.**

| Plasmid | Description or relevant characteristics[a] | Source |
|---|---|---|
| **Cloning and helper plasmids** | | |
| pJN105 | Broad host range cloning vector with an *E. coli araC*-$P_{BAD}$ controlled expression cassette | 9 |
| pDONR221 | Gateway DONR vector with *ccdB* toxin and *cat* flanked by *attP1* and *attP2* sites, $Kn^r$, $Cm^r$ | Invitrogen |
| pTNS2 | Vector for expressing TnsABCD, used in miniTn7 mutagenesis | 10 |
| pFLP2 | Vector for expression of Flp recombinase, encodes *sacB* for counterselection, $Ap^r$ | 11 |
| **Promoter-reporter fusions** | | |
| pMH487 | pUCP22Not-RNase III-*gfp*(Mut3)-$T_0$-$T_1$, $Ap^r$, $Gm^r$ | 12 |
| pCdrA::*gfp*$^S$ | pUCP22Not-$P_{cdrA}$-RBS-CDS-RNaseIII-*gfp*(Mut3)-$T_0$-$T_1$, $Ap^r$, $Gm^r$ | 12 |
| pMS402 | Promoter-less *luxCDABE* reporter, $Kn^r$ | 13 |
| pMS402-$P_{cdrA}$-*luxCDABE* | pMS402 with $P_{cdrA}$-*luxCDABE*, $Kn^r$ | 14 |
| **Allelic exchange vectors** | | |
| pEX18Ap | allelic exchange vector, $Ap^r$ | 11 |
| pLS6 | pEX18Ap with the *tdcA*$^{162\Delta G}$ allele from *P. aeruginosa* CF39, $Ap^r$ | This study |
| pLS11 | pEX18Ap with the *tdcA* allele from *P. aeruginosa* CF39S, $Ap^r$ | This study |
| pDONORPEX18Gm | allelic exchange vector, $Gm^r$, $Cm^r$ | 5 |
| pTER124D | pDONORPEX18Gm::Δ*pelF*::$P_{01/03/04}$-RBSII-mCherry, $Gm^r$ | This study |
| pDONRPEX18Gm-GFPmut3* | dual-marker allelic exchange vector, expresses GFPmut3*, $Gm^r$, $Cm^r$ | This study |
| pTER125A | pDONORPEX18Gm-$P_{01/03/04}$-RBSII-GFPmut3*::Δ*pslD*, $Gm^r$ | This study |
| pTER150E | pDONORPEX18Gm-$P_{01/03/04}$-RBSII-GFPmut3*::Δ*fleQ*, $Gm^r$ | This study |
| **miniTn7 and miniCTX vectors for site-directed chromosomal insertions** | | |
| pUC18-miniTn7T-Gm | pUC18 containing the miniTn7 transposon, $Gm^r$, $Ap^r$ | 10 |
| pUC18T-miniTn7T-Gm | pUC18 containing the miniTn7 transposon, mobilizable, $Gm^r$, $Ap^r$ | 10 |

| | | |
|---|---|---|
| pDONRPUC18- miniTn7T2.1-Gm | pUC18-miniTn7T-Gm with terminators flanking each side of a Gateway® donor site, $Gm^r$, $Ap^r$, $Cm^r$ | Lab archive |
| pUC18-miniTn7T2.1-Gm-GW | pUC18-miniTn7T-Gm with terminators flanking each side of a Gateway® destination site, $Gm^r$, $Ap^r$, $Cm^r$ | [6] |
| pBT270 | pUC18-miniTn7T2.1-Gm-GW::$P_{01/04/03}$-*gfp*mut3*, $Ap^r$, $Gm^r$ | [6] |
| pBT277 | pUC18-miniTn7T2.1-Gm-GW::$P_{01/04/03}$-mCherry, $Ap^r$, $Gm^r$ | [6] |
| pTER1 | pDONR221 encoding the putative promoter of the *tdcA* operon fused to the *tdcA* allele from CF39S, $Kn^r$ | This study |
| pKL1 | pDONR221 encoding the putative promoter of the *tdcA* operon fused to the $tdcA_{162\Delta G}$ allele from CF39, $Kn^r$ | This study |
| pJJH293 | pDONR221 with a 7051 bp fragment encoding the predicted 7-gene *tdcA* operon from *P. aeruginosa* CF39S, $Kn^r$ | This study |
| pKL2 | pDONR221 with a 7051 bp fragment encoding the predicted 7-gene $tdcA_{162\Delta G}$ operon from *P. aeruginosa* CF39, $Kn^r$ | This study |
| pHA215 | pUC18-miniTn7T2.1-Gm-GW encoding the putative 7-gene *tdcA* operon from CF39S, $Ap^r$, $Gm^r$ | This study |
| pKL3 | pUC18-miniTn7T2.1-Gm-GW encoding the putative 7-gene $tdcA_{162\Delta G}$ operon from CF39, $Ap^r$, $Gm^r$ | This study |
| pTER9 | pUC18-miniTn7T2.1-Gm-GW encoding the putative promoter of the *tdcA* operon fused to *tdcA* from CF39S, $Ap^r$, $Gm^r$ | This study |
| pKL4 | pUC18-miniTn7T2.1-Gm-GW encoding the putative promoter of the *tdcA* operon fused to $tdcA_{162\Delta G}$ from CF39, $Ap^r$, $Gm^r$ | This study |
| pHA208 | pUC18T-miniTn7T-Gm*::araC-*$P_{BAD}$*::tdcA*, $Ap^r$, $Gm^r$ | This study |
| pHA210 | pUC18T-miniTn7T-Gm*::araC-*$P_{BAD}$*::stp1*, $Ap^r$, $Gm^r$ | This study |
| pHA211 | pUC18T-miniTn7T-Gm*::araC-*$P_{BAD}$*::wspR*$^{V72D}$, $Ap^r$, $Gm^r$ | This study |
| pDONR221::$tdcA_{Ga}$ | pDONR221 with a synthetic gene corresponding to the TdcA homolog from *Gallionellales* bacterium GWA2_54_124 | Invitrogen |
| pHA245 | pUC18-miniTn7T2.1-Gm*::araC-*$P_{BAD}$*::* $tdcA_{Ga}$, $Ap^r$, $Gm^r$ | This study |
| miniCTX2 | Integration proficient vector, $Tc^r$ | [15] |
| **Protein production vectors** | | |
| pHMGWA | Destination vector derived from pET22b, contains N-terminal His×6 and maltose binding protein (MBP) tags, $Ap^r$ | [16] |
| pHNGWA | Destination vector derived from pET22b, contains N-terminal His×6 and N-utilizing substance (NusA) tags, $Ap^r$ | [16] |
| pJJH295 | pDONR221 bearing *tdcA* with an N-term TEV tag, $Kn^r$ | This study |
| pJGR10 | pDONR221 bearing *dosP*, $Kn^r$ | This study |

| | | |
|---|---|---|
| pAraCpBAD/His6/lacZ | Vector for expression of recombinant *E. coli* $His_6$-LacZ, arabinose inducible, $Ap^r$ | R. Eugene Huber |
| pTER132 | Vector for expression of recombinant *E. coli* LacZ- $His_6$, arabinose inducible, $Ap^r$ | This study |
| pTER143 | Vector for expression of recombinant thermo-gal ($LacZ^{1-92}$-$TdcA^{1-177}$-$LacZ^{93-1090}$-$His_6$), arabinose inducible, $Ap^r$ | This study |
| pTER145 | Vector for expression of recombinant $LacZ^{\Delta 4-41}$-$His_6$, arabinose inducible, $Ap^r$ | This study |
| pEA03-A | Vector for expression of recombinant PAS2-gal ($LacZ^{1-92}$-$PdeO^{2-138}$-$LacZ^{93-1090}$-$His_6$), arabinose inducible, $Ap^r$ | This study |
| pJJH319 | pHMGWA destination vector for expression of TEV-TdcA with N-terminal His×6 and maltose binding protein (MBP) tags, $Ap^r$ | This study |
| pJJH320 | pHNGWA destination vector for expression of TEV-TdcA with N-terminal His×6 and N-utilizing substance protein A (NusA) tags, $Ap^r$ | This study |
| pJGR11 | pHMGWA destination for expression of DosP with N-term His×6 and maltose binding protein (MBP) tags, $Ap^r$ | This study |
| pETduet::wspR/PA2133 | Protein production vector for WspR, $Ap^r$, $Cm^r$ | [17] |
| pETduet::$wspR_{V72D}$/PA2133 | Protein production vector for $WspR_{V72D}$, $Ap^r$, $Cm^r$ | This study |
| pTER90 | pJJH319 with a site directed mutation enabling the expression of $His_6$-MBP-$TdcA^{E257A}$, $Ap^r$ | This study |
| pTER91 | pJJH319 with a site directed mutation enabling the expression of $His_6$-MBP-$TdcA^{M1*}$ (i.e. $His_6$-MBP), $Ap^r$ | This study |
| pTER92 | pJJH319 with a site directed mutation enabling the expression of $His_6$-MBP-$TdcA^{M167*}$ (i.e. $His_6$-MBP-$PAS3_{TdcA}$), $Ap^r$ | This study |

[a] Abbreviations for antibiotic selection: Ap, ampicillin; Cb, carbenicillin; Cm, chloramphenicol, Gm = Gentamycin; Tc, tetracycline.

**Supplementary Table 3. Primers.**

| Oligonucleotide | DNA sequence |
|---|---|
| **DNA Sequencing and PCR detection of mutant alleles** | |
| oJJH367_M13F | GTA AAA CGA CGG CCA G |
| oJJH368_M13R | CAG GAA ACA GCT ATG AC |
| oJJH1695_miniTn7F01-Seq | CTA ATT CGA TCA TGC ATG AGC TC |
| oJJH1696_miniTn7R02-Seq | CAC TTA TCT GGT TGG CCT GCA AG |
| oJGR187 | CGC ATG TTT CGC TGT TC |
| oJGR188 | GCG AAG ATT TGC GGC TG |
| oJGR161 | *TCG AGG TCG ACG GTA TCG ATA AGC TT*A TGA AGC TAA CCG ATG CG |
| oJJH1906_T7term | GCT AGT TAT TGC TCA GCG G |
| oKMC179_pslDF1 | CCG AGG TCT ACC ATT CCC ACG |
| oKMC180_pslDR1 | GAA CTT GGT GCG CTT CCA CAG |
| oKMC181_pelFF1 | CTG GTA CTG GGA ACT GGC CTA CC |
| oKMC182_pelFR1 | CAC GCT GAC GAT CGA CAG CAC |
| oTER056 | GCG CCC TTG TCT TTT TGC CTG |
| oTER057 | GTG CGA GTA CCG CGT TCG TC |
| oTER058 | CGG TTC GAC AAG CCG GTC AAG |
| oTER092 | GAT CCA GCA CCC AAA TCC AAA CG |
| oTER277 | CAT TGA TTA TTT GCA CGG CG |
| oTER278 | ACT ACC ATC GGC GCT AC |
| oTER279 | GAG TGA CGG CAG TTA TCT G |
| oTER280 | GCG AAT GGT GCA GCG C |
| oTER281 | GAA GTG CCT CTG GAT GTC |

| | |
|---|---|
| oTER282 | GCA GGT AGC AGA GCG G |
| oTER283 | GAT GTG CTG CAA GGC G |
| oTER284 | GCG GTC AGG GGG TGG |
| oTER306 | CGA AAG TAA ACC CAC TGG |
| oTER307 | AGG GTC AAT GCG GGT C |

**Construction of allelic exchange vectors**

| | |
|---|---|
| oLS14 | ATC C**GG ATC C**AT GAA CGG GCC GAC CAT C |
| oLS15 | ATC C**CT GCA G**TC AAT TCT GAA ATA CTT GTG C |
| oJJH1435 | **GGG GAC AAG TTT GTA CAA AAA AGC AGG CTA** CGC TGG TAC TGG GAA CTG GC |
| oJJH1436 | *GCA ATC TCC GTG GCT TCG CGG TAC AG*C GGA GCG GTG TGT TCG GTC |
| oJJH1437 | CTG TAC CGC GAA GCC ACG G |
| oTER230 | GGG GA**G AGC TC**C CAT CAG AAA ATT TAT CAA AAA GAG TG |
| oTER231 | GGG GA**G AGC TC**C AGG CTC GGC TTA TTT GTA TAG |
| oTER232 | *CAC TCT TTT TGA TAA ATT TTC TGA TGG G*AC AGG GTC GCC AGC AAT ATC G |
| oTER233 | CCC ATC AGA AAA TTT ATC AAA AAG AGT G |
| oTER234 | **GGG GAC CAC TTT GTA CAA GAA AGC TGG** GCA GGC TCG TCG AAT TCT TAC |
| oTER235 | *CAG GAA GTG CTC CCT CAT GAA A*CG CTG AGG AGC GAC ATC GCC ATG ATA G |
| oTER236 | *CTA TCA TGG CGA TGT CGC TCC TCA* GCG TTT CAT GAG GGA GCA CTT CCT G |
| oTER237 | **GGG GAC AAG TTT GTA CAA AAA AGC AGG CTA** CAG CAA GCG CCT GGC CGA C |
| oTER238 | **GGG GAC CAC TTT GTA CAA GAA AGC TGG** GTG ATC TCC ATC ACC GTC GAG |
| oTER437 | **GGG GAC AAG TTT GTA CAA AAA AGC AGG CTT** AAA CCG GTA GCG CCC TG |
| oTER438 | GGT TTC GCG CCA CAT TTT G |
| oTER439 | *GGC AGC TGA TCA AAA TGT GGC GCG AAA CC*G ATG ATT GAC AGG TCG TTT C |
| oTER440 | **GGG GAC CAC TTT GTA CAA GAA AGC TGG** GCG TCG CGC AGG GAA ATC |

| | |
|---|---|
| oTER464 | GCT CGA GGA AGT TTC CG |
| oTER465 | GGT GAG GTC GTT GAG C |
| oTER240 | GCT CAC TCA TTA GGC ACC C |
| oTER241 | GCA GGT CGA CTC TAG AGG |
| oTER242 | CTA GAG TCG ACC TGC |
| oTER247 | ACA ATA GCG AGT AGG CTG G |
| oTER5 | ATG CGT AAA GGA GAA GAA CTT TTC |

**Construction of miniTn7 vectors (and corresponding Gateway® entry plasmids)**

| | |
|---|---|
| oJJH1213 | **GGG GAC AAG TTT GTA CAA AAA AGC AGG CTC** GCT TGG CTG CTT CGC TGT G |
| oJJH1218 | **GGG GAC CAC TTT GTA CAA GAA AGC TGG GTA** GTA AGG GCG TGA AAA ACC AAA CC |
| oTER1 | **GGG GAC AAG TTT GTA CAA AAA AGC AGG CTT** ACA TAT CCA ATC ACC CGG GTC C |
| oTER2 | *GAC TGA CTG AGT GCC TGC CGT AAT TC*A GCG GGT TCA ACC GCT TTT TC |
| oTER3 | GAA TTA CGG CAG GCA CTC AG |
| oTER11 | **GGG GAC CAC TTT GTA CAA GAA AGC TGG GTC** TCA ATT CTG AAA TAC TTG TGC ACC |
| oHA11_wspR_GGDEF_SOE | CTG ACC GGG CTC TCC AAC CGT CGT |
| oHA12_wspR_GGDEF_attB2 | **GGG GAC CAC TTT GTA CAA GAA AGC TGG GTA** TCA GCC CGC CGG GGC CGG |
| oHA13_tdcA_PAS_attB1 | **GGG GAC AAG TTT GTA CAA AAA AGC AGG CTC A**AT GAA CGG GCC GAC CAT CTG GCT G |
| oHA14_tdcA_PAS_SOE | *ACG ACG GTT GGA GAG CCC GGT CAG* GGG GTC GGT GAT CGC CAT |
| oHA229_miniTn7_fwr_pBAD | *GAT CCC CCG GGC TGC AGG AAT TCC TCG AGA* TTA TGA CAA CTT GAC GGC TAC AT |
| oHA230_miniTn7_rev_pBAD | AGT GGA TCC CCC GGG CTG CAG GAA TTC GCT |
| oHA231_miniTn7_fwr_wspR | *AGC GAA TTC CTG CAG CCC GGG GGA TCC ACT* ATG CAC AAC CCT CAT GAG AGC AAG ACC GAC |
| oHA232_miniTn7_rev_wspR | *TGG TTG GCC TGC AAG GCC TTC GCG AGG TAC TCA* GCC CGC CGG GGC CGG CGG CAC CGG CTG |
| oHA233_miniTn7_rev_tdcA | *TGG TTG GCC TGC AAG GCC TTC GCG AGG TAC TCA* ATT CTG AAA TAC TTG TGC ACC CAC A |
| oHA234_miniTn7_fwr_tdcA | *AGC GAA TTC CTG CAG CCC GGG GGA TCC ACT* ATG AAC GGG CCG ACC ATC TGG CTG ATG |

| | |
|---|---|
| oHA235_miniTn7_fwr_stp1 | *AGC GAA TTC CTG CAG CCC GGG GGA TCC ACT* ATG AAC GGG CCG ACC ATC TGG CTG ATG GTG |
| oHA236_miniTn7_rev_stp1 | TGG TTG GCC TGC AAG GCC TTC GCG AGG TAC TCA GCC CGC CGG GGC CGG CGG CAC CGG CTG |
| oHA274_ miniTn7_tdcAga_fwr | *AGC GAA TTC CTG CAG CCC GGG GGA TCC ACT* ATG ATG GAT TTT TTC CTG AAT CTG |
| oHA275_ miniTn7_tdcAga_rev | *TGG TTG GCC TGC AAG GCC TTC GCG AGG TAC TCA* TTA AGC CGC ATC AAA CCG TTG |
| oHA237_pBAD-dgc_fwr | CCG GGG GAT CCA CTA T |
| oHA238_pBAD-dgc_rev | GCA AGG CCT TCG CGA GG |
| **Protein expression vectors** | |
| oJJH1550_tdcAR01-attB2 | **GGG GAC CAC TTT GTA CAA GAA AGC TGG GTC** TCA ATT CTG AAA TAC TTG TGC ACC |
| oJJH1692_tdcAF02-attB1-TEV | **GGG GAC AAG TTT GTA CAA AAA AGC AGG CT**T CGA AAA CCT GTA CTT CCA GGG CAT GAA CGG GCC GAC CAT CTG |
| oJGR182_DosPF1 | **GGG GAC AAG TTT GTA CAA AAA AGC AGG CTC** AAT GAA GCT AAC CGA TGC G |
| oJGR183_DosPR1 | **GGG GAC CAC TTT GTA CAA GAA AGC TGG GTA** TCA GAT TTT CAG CGG TAA C |
| oTER285 | *CGC CAG TGT GCT GGA ATT CGG CTT GTT C*GA AAC GAT GAT AGA TCC CGT CG |
| oTER286 | *CGA ACA AGC CGA ATT CCA GCA CAC TGG CG*G CCG TTA CTA GTG GAT CCA TGG TTA ATT CCT CCT G |
| oTER287 | *CCG CAA GCC GAA TTC TGC AGA TAT CC*A TCA CAC TGG CGG CCG CTC GAG ATC TGC AGC TGG TAC CAT ATG GGA ATT CGA AGC TTG GGC CCG AAC AAA AAC TCA TCT CAG AAG AGG ATC TGA ATA GCG CCG TCG ACC ATC ATC ATC ATC ATC ATT GA*G TTT AAA CGG TCT CCA GCT TGG CTG TTT TGG C* |
| oTER288 | *GCC AAA ACA GCC AAG CTG GAG ACC GTT TAA AC*T CAA TGA TGA TGA TGA TGA TGG TCG ACG GCG CTA TTC AGA TCC TCT TCT GAG ATG AGT TTT TGT TCG GGC CCA AGC TTC GAA TTC CCA TAT GGT ACC AGC TGC AGA TCT CGA GCG GCC GCC AGT GTG ATG *GAT ATC TGC AGA ATT CGG CTT GCG G* |
| oTER289 | *GGA TAT CTG CAG AAT TCG GCT TGC GG*C CGC TTT TTG ACA CCA GAC CAA CTG |
| oTER290 | *GTT TAA ACG GTC TCC AGC TTG GCT GTT TTG GC*G GAT GAG AGA AGA TTT TC |
| oTER305 | *GTA TCA GGC TGA AAA TCT TCT CTC ATC* CGC CAA AAC AGC CAA GCT GGA GAC C |
| oTER322 | *CCC TCA AAC TGG CAG ATG CAC GGT TAC GAT* ATG AAC GGG CCG ACC ATC |
| oTER323 | *CCA TCA GCC AGA TGG TCG GCC CGT TCA T*AT CGT AAC CGT GCA TCT G |
| oTER324 | *GCA GCG GTT GCT GCG CAT GGC GAT CAC C*GC GCC CAT CTA CAC C |
| oTER325 | *GGG ATA GGT CAC GTT GGT GTA GAT GGG CG*C GGT GAT CGC CAT GCG |

| | |
|---|---|
| oTER329 | *GGG ATA GGT CAC GTT GGT GTA GAT GGG CGC* ATC TAT CAT CGT TTC GAA CAA GC |
| oTER411 | ATC GTA ACC GTG CAT CTG |
| oTER412 | GCG CCC ATC TAC ACC |
| oTER413 | *CCC TCA AAC TGG CAG ATG CAC GGT TAC GAT* AAG CTA ACC GAT GCG GA |
| oTER414 | *GGG ATA GGT CAC GTT GGT GTA GAT GGG CGC* CAA TTG CCT GGT CTG TTC T |
| **Site-directed mutagenesis** | |
| oTER269_wspRV72D | ATC CTC CAG GAC CTG GAT ATG CCC GGC GTC GAC |
| oTER270_wspRV72D | GTC GAC GCC GGG CAT ATC CAG GTC CTG GAG GAT |
| oTER047 | GGA TTG GGG GGG CGG AAT TCG CTG TG |
| oTER048 | CAC AGC GAA TTC CGC CCC CCC AAT CC |
| oTER170 | CTG TAC TTC CAG GGC TAA AAC GGG CCG ACC ATC |
| oTER171 | GAT GGT CGG CCC GTT TTA GCC CTG GAA GTA CAG |
| oTER172 | CTT CTC GCA AGG AAT AAG AGC AGC GGT TGC TG |
| oTER173 | CAG CAA CCG CTG CTC TTA TTC CTT GCG AGA AG |

**Italics* denote a region of complementarity for SOE-PCR or Gibson assembly; underlined sequence denotes a region specific to the targeted DNA amplicon; **bold** denotes a restriction site, *attB1* or *attB2* sequence.

**Supplementary References**

1 Jones, P. *et al.* InterProScan 5: genome-scale protein function classification. *Bioinformatics* **30**, 1236-1240, (2014).
2 Starkey, M. *et al.* *Pseudomonas aeruginosa* rugose small colony variants have adaptations that likely promote persistence in the cystic fibrosis lung. *J. Bacteriol.* **191**, 3492-3503, (2009).
3 Jacobs, M. A. *et al.* Comprehensive transposon mutant library of *Pseudomonas aeruginosa*. *Proc. Natl. Acad. Sci. U. S. A.* **100**, 14339-14344, (2003).
4 Harrison, J. J. *et al.* Elevated exopolysaccharide levels in *Pseudomonas aeruginosa* flagellar mutants have implications for biofilm growth and chronic infections. *PLoS Genet.* **16**, e1008848, (2020).
5 Hmelo, L. R. *et al.* Precision-engineering the *Pseudomonas aeruginosa* genome with two-step allelic exchange. *Nat. Protoc.* **10**, 1820-1841, (2015).
6 Zhao, K. *et al.* Psl trails guide exploration and microcolony formation in *Pseudomonas aeruginosa* biofilms. *Nature* **497**, 388-391, (2013).
7 Almblad, H. *et al.* The cyclic AMP-Vfr signaling pathway in *Pseudomonas aeruginosa* is inhibited by cyclic di-GMP. *J. Bacteriol.* **197**, 2190-2200, (2015).
8 Simon, R., Priefer, U. & Pühler, A. A broad host range mobilization system for *in vivo* genetic engineering: transposon mutagenesis in gram negative bacteria. *Nat. Biotechnol.* **1**, 784-791, (1983).
9 Newman, J. R. & Fuqua, C. Broad-host-range expression vectors that carry the l-arabinose-inducible *Escherichia coli araBAD* promoter and the *araC* regulator. *Gene* **227**, 197-203, (1999).
10 Choi, K. H. & Schweizer, H. P. mini-Tn7 insertion in bacteria with single attTn7 sites: example *Pseudomonas aeruginosa*. *Nat. Protoc.* **1**, 153-161, (2006).
11 Hoang, T. T., Karkhoff-Schweizer, R. R., Kutchma, A. J. & Schweizer, H. P. A broad-host-range Flp-FRT recombination system for site-specific excision of chromosomally-located DNA sequences: application for isolation of unmarked *Pseudomonas aeruginosa* mutants. *Gene* **212**, 77-86, (1998).
12 Rybtke, M. T. *et al.* Fluorescence-based reporter for gauging cyclic di-GMP levels in *Pseudomonas aeruginosa*. *Appl. Environ. Microbiol.* **78**, 5060-5069, (2012).
13 Duan, K. & Surette, M. G. Environmental regulation of *Pseudomonas aeruginosa* PAO1 Las and Rhl quorum-sensing systems. *J. Bacteriol.* **189**, 4827, (2007).
14 Borlee, B. R., Borlee, G. I., Martin, K. H. & Irie, Y. Cyclic di-GMP-responsive transcriptional reporter bioassays in *Pseudomonas aeruginosa*. *Methods Mol. Biol.* **1657**, 99-110, (2017).
15 Hoang, T. T., Kutchma, A. J., Becher, A. & Schweizer, H. P. Integration proficient plasmids for *Pseudomonas aeruginosa*: site-specific integration and use for engineering of reporter and expression strains. *Plasmid* **43**, 59-72, (2000).
16 Busso, D., Delagoutte-Busso, B. & Moras, D. Construction of a set Gateway-based destination vectors for high-throughput cloning and expression screening in *Escherichia coli*. *Anal. Biochem.* **343**, 313-321, (2005).
17 Huangyutitham, V., Guvener, Z. T. & Harwood, C. S. Subcellular clustering of the phosphorylated WspR response regulator protein stimulates its diguanylate cyclase activity. *mBio* **4**, (2013).
