# Supplementary material for: Bacterial cyclic diguanylate signaling networks sense temperature
Source: Nat Commun. 2021 Mar 31;12:1986. doi: 10.1038/s41467-021-22176-2 (PMC8012707; doi:10.1038/s41467-021-22176-2)
Supplement: Supplementary file 3 — Description of Additional Supplementary Files [file 41467_2021_22176_MOESM3_ESM.pdf]

Description of Additional Supplementary Files

File Name: Supplementary Movie 1
Description: *Time-lapse imaging of bacterial thermotransduction. Here, Pseudomonas fluorescens Pf-81 was engineered to express TdcA and a luminescent c-di-GMP bioreporter. Bacteria were grown on LB agar at 25 °C and then shifted to an incubator at 37 °C. Strains denoted tdcA- have the* $tdcA_{162\Delta G}$ *allele, and VC denotes the promoterless, vector control for the luxCDABE bioreporter.*

File Name: Supplementary Data 1
Description: *Lists of TdcA orthologs from P. aeruginosa and its homologs from other bacterial species.*
