# Supplementary material for: Bacterial cyclic diguanylate signaling networks sense temperature
Source: Nat Commun. 2021 Mar 31;12:1986. doi: 10.1038/s41467-021-22176-2 (PMC8012707; doi:10.1038/s41467-021-22176-2)
Supplement: Supplementary file 6 — Reporting Summary [file 41467_2021_22176_MOESM6_ESM.pdf]

nature research

Corresponding author(s): Dr. Joe Jonathan Harrison

Last updated by author(s): 01/31/2021

Reporting Summary

Nature Research wishes to improve the reproducibility of the work that we publish. This form provides structure for consistency and transparency in reporting. For further information on Nature Research policies, see our Editorial Policies and the Editorial Policy Checklist.

Statistics

For all statistical analyses, confirm that the following items are present in the figure legend, table legend, main text, or Methods section.

n/a	Confirmed	
☐	☒	The exact sample size (*n*) for each experimental group/condition, given as a discrete number and unit of measurement
☐	☒	A statement on whether measurements were taken from distinct samples or whether the same sample was measured repeatedly
☐	☒	The statistical test(s) used AND whether they are one- or two-sided<br>*Only common tests should be described solely by name; describe more complex techniques in the Methods section.*
☒	☐	A description of all covariates tested
☒	☐	A description of any assumptions or corrections, such as tests of normality and adjustment for multiple comparisons
☐	☒	A full description of the statistical parameters including central tendency (e.g. means) or other basic estimates (e.g. regression coefficient) AND variation (e.g. standard deviation) or associated estimates of uncertainty (e.g. confidence intervals)
☐	☒	For null hypothesis testing, the test statistic (e.g. *F*, *t*, *r*) with confidence intervals, effect sizes, degrees of freedom and *P* value noted<br>*Give P values as exact values whenever suitable.*
☒	☐	For Bayesian analysis, information on the choice of priors and Markov chain Monte Carlo settings
☒	☐	For hierarchical and complex designs, identification of the appropriate level for tests and full reporting of outcomes
☒	☐	Estimates of effect sizes (e.g. Cohen's *d*, Pearson's *r*), indicating how they were calculated

Our web collection on statistics for biologists contains articles on many of the points above.

Software and code

Policy information about availability of computer code

Data collection	Digital images of bacterial colonies were collected using NIS Elements® v.4.13.00. Images of gels, Western blots and luminescent bacteria on agar plates were captured using Alphaview (v3.4.0) (Proteinsimple).
Data analysis	Primer design was carried out using SnapGene Viewer (v2.0 up to v4.3.10, updated periodically) or Geneious (Geneious Prime 2020.1, Geneious Prime 2019.2, Geneious R11.1, Geneious R10.2, Geneious R9.1, Geneious R8.1.9, Geneious R7.1.9, updated periodically during execution of the research). Bioinformatics databases were built in Geneious. Sequence Alignments were carried out using Clustal Omega (v1.2.2), and PSIPRED (v4.0). Alignments for phylogenetic analysis were done using MUSCLE (v3.8.425). Phylogenetic sequence analysis was carried out using FastTree2 (v2.1.12) and InterProScan (v1.1.4) plugins as described in Methods. Genome assembly and variant analysis were carried out using previously published bioinformatic pipelines as described in Methods. The specific software for the sequence analysis, assembly, comparative genomics, and genome annotation are as follows: Newbler (v2.0.22.19), SMRT Analysis for PacBio® RSII (v.2.3.0), MapSolver® (v3.2.0), Prokka (v.1.12), DIAMOND (v1), Island Viewer (v4), Resistance Gene Identifier (RGI) tool (v4.2.2), and PSORTb (v3.0). Mass spec data was analyzed using MAVEN (v8.1.25.3). Graphing and statistical analyses were executed in GraphPad Prism 7 (v7.02 and v7.03). Figures were assembled using Adobe Illustrator (CS3, CS5, CC 2018 Subscription) and Photoshop Creative Suite (CS3, CS5, CC 2018 Subscription). Images were assembled into a movie (for Supplementary Movie 1) using iMovie v10.2.2.

For manuscripts utilizing custom algorithms or software that are central to the research but not yet described in published literature, software must be made available to editors and reviewers. We strongly encourage code deposition in a community repository (e.g. GitHub). See the Nature Research guidelines for submitting code & software for further information.

Data

Policy information about availability of data

All manuscripts must include a data availability statement. This statement should provide the following information, where applicable:

- Accession codes, unique identifiers, or web links for publicly available datasets
- A list of figures that have associated raw data
- A description of any restrictions on data availability

Finished CF39S and pCF39S nucleotide sequences were deposited in the Pseudomonas genome database (www.pseudomonas.com), and are also available through NCBI GenBank with Accession Numbers NZ_CP045916.1 (https://www.ncbi.nlm.nih.gov/nuccore/NZ_CP045916.1) and NZ_CP045917 (https://www.ncbi.nlm.nih.gov/nuccore/NZ_CP045917), respectively. Source data are provided with this paper. Data plotted in Figs. 1b, 1c, 1e, 2c, 2d, 2e, Fig. 3b-f, and 4b as well as Supplementary Figures 1, 3, and 4-6 are provided in the Source Data. Unedited, original images and replicates of Western blots shown in Fig. 2 appear in Supplementary Figures 8-10. Sequences and metadata used to generate Supplementary Figures 2 and 3 are provided in Supplementary Data 1. All bacterial strains and plasmids are available from the corresponding author on request.

Field-specific reporting

Please select the one below that is the best fit for your research. If you are not sure, read the appropriate sections before making your selection.

☒ Life sciences ☐ Behavioural & social sciences ☐ Ecological, evolutionary & environmental sciences

For a reference copy of the document with all sections, see nature.com/documents/nr-reporting-summary-flat.pdf

Life sciences study design

All studies must disclose on these points even when the disclosure is negative.

Sample size	No sample size calculation was performed. However, all measurements were conducted in biological triplicate or greater, which is a standard of practice in molecular microbiological and biochemical research. Multiple technical replicates were performed for each biological replicate in high-throughput biofilm assays carried out in microplates (6 or greater technical replicates each). Mass spectrometry measurements of the intracellular second messenger cyclic diguanylate (c-di-GMP) were performed in three to twelve biological replicates to account for intrinsic variation of the technique.
Data exclusions	The Grubs statistical test (Alpha of 0.05) was used to identify and eliminate outliers from mass spectrometry datasets that may have resulted from short-term, run-to-run instrumental variance (applied to data in Fig. 1c, Fig. 3f, Fig. 4b, and Supplementary Figures 1 and 3c). Spectrophotometric measurements in biofilm microplate assays were excluded from the analysis if the background-corrected measurement was less than zero (i.e. below the threshold of detection in the experimental method; applied to data in Fig. 1b, 1e, and 2e). Spectrophotometric measurements for hemin less than 280 nm were excluded from Supplementary Figure 6 due to high background absorbance from ethanol that was required to dissolve hemin. No other data points were excluded from any analysis.
Replication	All attempts at replication were successful. Biological replicates were performed on separate days and/or on bacterial cultures propagated from distinct starter cultures. Reproducibility of experimental findings was additionally verified by having different individuals perform the same experimental measurements independently. A representative technical replicate for the digunylate cyclase activity of the His6-NusA-TdcA fusion is shown in Fig. 2b; however, the data for this activity were verified and reproduced by showing similar catalytic activities for a His6-MBP-TdcA fusion in vitro (ex. Fig. 2c, 2e and 2f). Supplementary Figure 7 contains a technically replicated, representative gel image from a single batch purification of protein from Escherichia coli, which was executed once as a qualitative standard practice for assessing recombinant protein expression levels in different strains.
Randomization	Positional effects of assays in microplates were controlled by varying positions of test and control groups in microplate wells. Further randomization of test and control organisms between groups does not apply to this research because this study employed engineered bacterial strains with known genotypes. Nevertheless, covariation in genotype that might confound results (which might be due to, for example, second site mutation on chromosomes independently of the engineered mutation) was controlled by performing complementation analysis. Additionally, genetic linkage analysis was executed to observe gain-of-function phenotypes in engineered heterologous organisms lacking the gene(s) of interest.
Blinding	Samples sent for mass spectrometry were numbered and technicians did not know the identity of samples being measured.

Reporting for specific materials, systems and methods

We require information from authors about some types of materials, experimental systems and methods used in many studies. Here, indicate whether each material, system or method listed is relevant to your study. If you are not sure if a list item applies to your research, read the appropriate section before selecting a response.

Materials & experimental systems

n/a	Involved in the study	
☐	☒	Antibodies
☒	☐	Eukaryotic cell lines
☒	☐	Palaeontology and archaeology
☒	☐	Animals and other organisms
☒	☐	Human research participants
☒	☐	Clinical data
☒	☐	Dual use research of concern

Methods

n/a	Involved in the study	
☒	☐	ChIP-seq
☒	☐	Flow cytometry
☒	☐	MRI-based neuroimaging

Antibodies

Antibodies used	Rabbit anti-PelC, anti-PelD and anti-PslG were provided by Dr. Lynne Howell at the Hospital for Sick Children in Toronto, ON, Canada. All primary antibodies were used at a dilution of 1:2500. Secondary, horseradish peroxidase-conjugated goat anti-rabbit IgG (H+L) (product number 31460, lot number: TI208014) were purchased from ThermoFisher Scientific. These secondary antibodies were used at a dilution of 1:25,000.
Validation	Rabbit anti-PelC, anti-PelD and anti-PslG were antibodies were internally validated using genetically engineered bacteria that lacked the gene for the target protein (negative controls) or that had a mutation that resulted in overproduction of the target protein (positive controls). Additional validation of antibodies was carried out by Dr. Howell in previous publications from her group. Secondary antibodies from a commercial supplier were validated by the manufacturer.
